# Supplementary material for: Role and mechanism of NCAPD3 in promoting malignant behaviors in gastric cancer
Source: Front Pharmacol. 2024 Apr 22;15:1341039. doi: 10.3389/fphar.2024.1341039 (PMC11070777; doi:10.3389/fphar.2024.1341039)
Supplement: Supplementary file 11 [file DataSheet2.ZIP › GSEA/GO_CC/my_analysis.Gsea.1599461408869/GO_SPLICEOSOMAL_COMPLEX.html]

Details for gene set GO\_SPLICEOSOMAL\_COMPLEX[GSEA]

|  || Dataset | filtered\_dataset.sample\_info.cls#WT\_versus\_NCAPD3\_MUT |
| Phenotype | sample\_info.cls#WT\_versus\_NCAPD3\_MUT |
| Upregulated in class | WT |
| GeneSet | GO\_SPLICEOSOMAL\_COMPLEX |
| Enrichment Score (ES) | 0.33979997 |
| Normalized Enrichment Score (NES) | 1.4008077 |
| Nominal p-value | 0.104166664 |
| FDR q-value | 0.5443584 |
| FWER p-Value | 0.999 |
Table: GSEA Results Summary

  

Fig 1: Enrichment plot: GO\_SPLICEOSOMAL\_COMPLEX      
 Profile of the Running ES Score & Positions of GeneSet Members on the Rank Ordered List

  

| SYMBOL | TITLE | RANK IN GENE LIST | RANK METRIC SCORE | RUNNING ES | CORE ENRICHMENT || 1 | 23450 | SF3B3 | 178 | 0.697 | -0.0351 | Yes |
| 2 | 55599 | RNPC3 | 227 | 0.652 | 0.0170 | Yes |
| 3 | 6426 | SRSF1 | 243 | 0.637 | 0.0909 | Yes |
| 4 | 5356 | PLRG1 | 338 | 0.573 | 0.0996 | Yes |
| 5 | 10291 | SF3A1 | 466 | 0.493 | 0.0739 | Yes |
| 6 | 9416 | DDX23 | 553 | 0.445 | 0.0713 | Yes |
| 7 | 6632 | SNRPD1 | 563 | 0.440 | 0.1233 | Yes |
| 8 | 58509 | CACTIN | 748 | 0.351 | 0.0379 | Yes |
| 9 | 27316 | RBMX | 762 | 0.342 | 0.0740 | Yes |
| 10 | 494115 | RBMXL1 | 763 | 0.342 | 0.1194 | Yes |
| 11 | 10492 | SYNCRIP | 779 | 0.334 | 0.1530 | Yes |
| 12 | 4670 | HNRNPM | 790 | 0.330 | 0.1897 | Yes |
| 13 | 9343 | EFTUD2 | 818 | 0.305 | 0.2108 | Yes |
| 14 | 26121 | PRPF31 | 825 | 0.301 | 0.2465 | Yes |
| 15 | 11157 | LSM6 | 830 | 0.296 | 0.2830 | Yes |
| 16 | 4691 | NCL | 843 | 0.270 | 0.3102 | Yes |
| 17 | 3312 | HSPA8 | 848 | 0.244 | 0.3398 | Yes |
| 18 | 9939 | RBM8A | 1093 | -0.474 | 0.2276 | No |
Table: GSEA details [plain text format]

  

Fig 2: GO\_SPLICEOSOMAL\_COMPLEX      
 Blue-Pink O' Gram in the Space of the Analyzed GeneSet

  

Fig 3: GO\_SPLICEOSOMAL\_COMPLEX: Random ES distribution      
 Gene set null distribution of ES for **GO\_SPLICEOSOMAL\_COMPLEX**
